# Plants respond to leaf vibrations caused by insect herbivore chewing

**DOI:** 10.1007/s00442-014-2995-6

**Published:** 2014-07-02

**Authors:** H. M. Appel, R. B. Cocroft

**Affiliations:** 1 Bond Life Sciences Center and Division of Plant Sciences, University of Missouri, 1201 East Rollins St., Columbia, MO 65211 USA; 2 Division of Biological Sciences, University of Missouri, Columbia, MO 65211 USA

**Keywords:** Vibrational signaling, Insect herbivore, Plant–insect interactions, Chemical defenses, Glucosinolates, Anthocyanins, *Arabidopsis thaliana*, *Pieris rapae*

## Abstract

Plant germination and growth can be influenced by sound, but the ecological significance of these responses is unclear. We asked whether acoustic energy generated by the feeding of insect herbivores was detected by plants. We report that the vibrations caused by insect feeding can elicit chemical defenses. *Arabidopsis thaliana* (L.) rosettes pre-treated with the vibrations caused by caterpillar feeding had higher levels of glucosinolate and anthocyanin defenses when subsequently fed upon by *Pieris rapae* (L.) caterpillars than did untreated plants. The plants also discriminated between the vibrations caused by chewing and those caused by wind or insect song. Plants thus respond to herbivore-generated vibrations in a selective and ecologically meaningful way. A vibration signaling pathway would complement the known signaling pathways that rely on volatile, electrical, or phloem-borne signals. We suggest that vibration may represent a new long distance signaling mechanism in plant–insect interactions that contributes to systemic induction of chemical defenses.

**Electronic supplementary material:**

The online version of this article (doi:10.1007/s00442-014-2995-6) contains supplementary material, which is available to authorized users.

## Introduction

The effects of sound on plant growth and other traits have been recognized for decades, but the ecological significance of these responses is unclear. While plant responses to wind and touch have been examined and have clear adaptive significance (Chehab et al. 2009), plant responses to acoustic energy have largely been studied in the absence of an ecological context. For example, there is a long tradition of exposing plants to musical sound (Klein and Edsall 1965; Telewski 2006; Jeong et al. 2004). Although music influences growth and germination in some plants, music contains such a wide range of frequencies, amplitudes and fine-temporal patterns that its usefulness as an experimental stimulus is limited. More systematic studies have found that some frequencies have a greater influence than others (Telewski 2006). For example, young roots of corn grow towards the source of continuous tones, transmitted as airborne or waterborne sound, and respond optimally to frequencies of 200–300 Hz (Gagliano et al. 2012a). While these studies bring us a step closer to being able to link plant responses to acoustic energy to ecologically relevant sound sources, the experimental stimuli still remain far removed from those produced by natural sources of acoustic energy in the plant’s environment.

One of the most relevant sources of acoustic energy in the immediate environment of a plant is the rich community of plant-associated arthropods, including herbivores, predators, and parasitoids (Cocroft and Rodriguez 2005). Plant-borne vibrations provide a wealth of information about the activities of insects on plants. Within the abundant arthropod community on plants, many ecological and social interactions depend on the perception and production of plant-borne vibrations (Hill 2008). Some 200,000 species of insects communicate using substrate vibrations to locate mates, attract mutualists, or exploit plant resources (Cocroft and Rodriguez 2005). Many more insects and other arthropods use such vibrations to locate prey or avoid predators (Barth 1998; Castellanos and Barbosa 2006; Casas and Magal 2006; Virant-Doberlet et al. 2011; Cocroft 2011). Chewing herbivores, in particular, produce characteristic, high-amplitude vibrations that travel rapidly to other parts of the plant. Predatory insects can use chewing vibrations to detect their prey from a considerable distance: for example, on soybean, the chewing vibrations of green clover worms elicited search by predatory stinkbugs 50 cm away (Pfannenstiel et al. 1995).

We suggest that the vibrations produced by chewing herbivores are an important source of acoustic energy for plants. If plants can detect and use this conspicuous, reliable and rapidly transmitted source of information about herbivore feeding, tissues far from the site of attack could use feeding vibrations to respond quickly to the threat of herbivory. A vibration signaling pathway would complement the known signaling pathways that rely on phloem-borne signals, airborne volatiles, or electrical signals (Wu and Baldwin 2009; Mousavi et al. 2013). Here we test the hypothesis that plant responses to herbivory, in the form of induced chemical defenses, can be elicited by the mechanical vibrations produced by chewing caterpillars. We report that *Arabidopsis thaliana* plants exposed to chewing vibrations produced greater amounts of chemical defenses in response to subsequent herbivory, and that the plants distinguished chewing vibrations from other environmental vibrations.

## Materials and methods

### Characterizing plant responses to herbivory: direct induction vs. priming, systemic vs. local responses

**Fig. 1:**
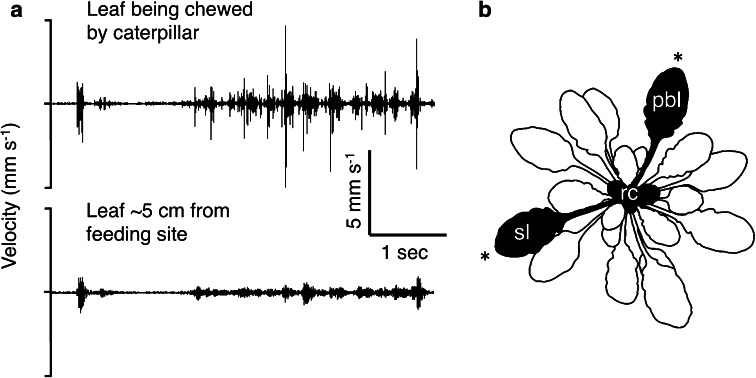
**a** Vibrations produced by a feeding *P. rapae* caterpillar on *A. thaliana*, recorded simultaneously (using two laser vibrometers) on the fed-upon leaf and a second leaf on the opposite side of the plant. These leaves correspond to the leaves labeled ‘pbl’ and ‘sl’ in the playback design shown in the next panel. **b** Sampling design for the experiments. An older leaf was selected for caterpillar recordings and vibrational playback (pbl), to allow attachment of an actuator with minimal effect on the rest of the plant. For the plants that experienced herbivory (all of the plants in experiment 1, and half of the plants in experiment 2), caterpillars were confined in clip cages placed on the playback leaf and a same-age leaf on the opposite side of the plant (sl). The two leaves experiencing herbivory 24 or 48 h after the experimental treatment are marked with an *asterisk*. The young unexpanded leaves in the rosette center (rc) were also sampled for leaf chemistry, but did not experience herbivory

Chemical defense responses can result from direct induction, such that the levels are higher after the initial herbivory or cue of herbivory; and/or they can be primed, such that levels of defense are higher or more rapid after subsequent herbivore attack (Frost et al. 2008). In the first experiment below, we sample leaves only after herbivory, allowing us to measure induced defenses elicited by vibration, but not to separate out direct effects vs. priming. In the second experiment, we include a no-herbivory treatment, allowing us to separate out priming from direct effects of chewing vibrations on plant responses. Induced responses can also be local, occurring only in tissues near the site of herbivory, or systemic, occurring over a larger spatial scale within the plant (Kessler and Baldwin 2002). Chewing vibrations are propagated rapidly to other leaves on the plant (Fig. 1a), and thus have the potential to trigger a systemic response. In the experiments below, we examine the potential for both local and systemic effects by sampling the leaf used for playback of chewing vibrations, a same-age leaf on the opposite side of the plant, and the unexpanded leaves in the rosette center.

### Plant growth

*A. thaliana* Col-0 plants were grown in individual #3 pots (55 × 57 mm) in potting soil (Pro-Mix; Premier Horticulture Inc., Quakertown, PA, USA) supplemented with 1.8 kg of Osmocote™ slow-release fertilizer (The Scotts Company, Marysville, OH) per cubic meter of soil. Plants were grown under metal halide lamps at 24 °C and 62 % relative humidity with a 8:16 h (L:D) 180 µmol m^−2^ s^−1^ photoperiod. Vegetative (rosette only) plants used in experiments were 4 weeks post-germination. Two days before the experiments, the plants were transplanted into 50-ml conical plastic centrifuge tubes to maximize the amount of leaf area overhanging the container to use for vibration treatment.

### Vibration recordings

To record caterpillar feeding vibrations, we allowed fourth-instar *P. rapae* caterpillars to feed on a leaf of a potted plant (*N* = 22 caterpillars and plants) and recorded the vibrations experienced by the fed-upon leaf, and a leaf on the opposite side of the plant (Fig. 1a, b). Chewing vibrations were recorded at 24.5 ± 1 °C with laser Doppler vibrometry (Polytec CLV 1000 and CLV M030 decoder module). To experimentally reproduce the caterpillar feeding vibrations, we used piezoelectric actuators supported under a leaf (Electronic Supplementary Material Fig. 1A) and attached to the leaf using accelerometer mounting wax. Before playback of the vibrations recorded on the fed-upon leaf, we characterized the frequency response of each actuator, then designed a digital filter that compensated for that response (Cocroft 2010). The playback stimuli were then filtered to yield playbacks that closely matched the temporal and spectral properties of the original recordings (Electronic Supplementary Material Fig. 2). We calibrated the amplitude of each playback to match that of the original recording.

We based our playback design on the feeding behavior of *P. rapae* caterpillars, which spend an average of 100 ± 223 min on a leaf, alternately feeding and resting (Coffman pers. comm.). Our playback stimuli consisted of 10 s of chewing followed by a 10 s pause, repeated for 5 min; there was then a 5 min pause. This basic 10-min pattern was repeated for 120 min to reflect the natural timing of *P.* *rapae* feeding activity.

### Insect growth and herbivory treatments

*P. rapae* (L.) were reared at 24 °C on *A. thaliana* plants grown in pots as described above, and are the progeny of biological stock originally obtained from Carolina Biological and the Jander laboratory (Cornell University, Ithaca, NY). Post-ecdysal fourth instar caterpillars were used for all experiments. Insects were removed from these plants for a maximum of 3 h before use. Caterpillars were placed on an older, outer rosette leaf like those used for vibrational playback, after which most individuals began feeding on the leaf. Laser vibrometry recordings of caterpillar feeding vibrations were made from the fed-upon leaf near the base of the leaf blade. The herbivory treatments were begun 30 min after playback of feeding vibrations. Individual larvae were confined using a clip cage (Electronic Supplementary Material Fig. 1B) to a fully expanded leaf in the rosette, and were allowed to feed until approximately 30 % of the leaf was removed. Leaves experiencing herbivory included the playback leaf and a same-age leaf on the opposite side of the plant (Fig. 1b). The no-herbivore treatment (Experiment #2, below) consisted of empty clip cages on corresponding leaves. Leaves were harvested into liquid N_2_ 24 and/or 48 h after caterpillar feeding, depending on the experiment.

### Defense chemistry

*A. thaliana* produces three major classes of chemical defenses in greater amounts following insect damage: glucosinolates (GSs: Mewis et al. 2005), the polyphenol anthocyanins (ACs: Ferrieri et al. 2013), and a suite of volatile compounds (Snoeren et al. 2010). Glucosinolate quantification procedures were adapted from previously described protocols (Mewis et al. 2005). Leaves were freeze-dried (2–4 mg DW) before being ground to a fine powder in a Talboys high throughput homogenizer (Troemer, NJ, USA) for extraction. Glucosinolates were extracted three times in 70 % methanol/DI H_2_O at 80 °C for 5 min. Supernatants were pooled and placed in a centrivap until dry. Pellets were re-suspended in 40 µL of 0.4 M barium acetate and 370 µL deionized water to precipitate protein, and desulphated overnight on DEAE Sephadex A-25 in 96-well filter plates. Plates were prepared by vacuum filtration with two 200-µL washes of 6 M imidazole formate followed by three additional washes of DI H_2_O. Crude glucosinolate extracts were added to individual wells and washed twice using sodium acetate buffer solution (pH 4.0). Sulfatase solution (30 µL) was added into to each sample for overnight desulfination at 4 °C. Desulfated glucosinolates were eluted twice in 150 µL of distilled water using a vacuum manifold. Detection and quantification of individual desulfated indolyl and aliphatic glucosinolates was performed using a Waters Alliance 2695 HPLC in tandem with a Waters Acquity TQ detector mass spectrometer, on a C18 RP column using a water/acetonitrile linear gradient. Glucosinolates were monitored by a UV detector at 229 nm and quantified using an internal standard (sinalbin) added prior to extraction. Our HPLC analyses allowed us to quantify the molar concentrations of ten individual glucosinolate compounds, including seven aliphatic glucosinolates [3-methylsulfinylpropyl (3 MSOP), 4-methylsulfinylbutyl (4MSOB), 5-methylsulfinylpentyl (5MSOP), 6-methylsulfinylhexyl (6MSOH), 7-methylsulfinylheptyl (7MSOH), 4-methylthiobutyl (4MTB), and 8-methylsulfinyloctyl (8MSOO)] and 3-indolyl glucosinolates [3-indoyl-methyl-(I3 M), 4-methoxy-3-indolylmethyl-(4MOI3 M), and 1-methoxy-3-indolylmethyl-(1MOI3 M)].

Polyphenols, including anthocyanins, were extracted and quantified as described previously (Ferrieri et al. 2013). Individual leaves were freeze-dried (4–6 mg DW), ground as described above, and extracted and quantified. Phenolics were extracted overnight in 200 μl of 1 % (v/v) HCl in methanol at 4 °C. An additional extraction with 250 μl distilled water and 500 μl chloroform was used to remove chlorophyll. Samples were vortexed and centrifuged for 3 min at 3,000×*g*. Relative anthocyanin levels in the aqueous phase were determined spectrophotometrically by measuring absorbance at 530 nm. Total flavonoid compounds were also estimated in the same extracts at absorbance 320 nm (Fukumoto and Mazza 2000; Shao et al. 2008). The concentration of total redox-reactive phenolics present in leaf extracts was determined using the Folin-Denis assay, with standard curves developed using chlorogenic and gallic acids, and standards purified from each treatment group (Appel et al. 2001).

### Experiment #1

In the first experiment, we played back caterpillar vibrations to naïve *A. thaliana* plants, using piezoelectric actuators (AE0505D18F actuators and MDT693B and MDR694B controllers, Thorlabs, Inc., Newton, New Jersey, USA). In each replicate (*N* = 22), we used a different chewing exemplar from the recordings made above. A single replicate included four plants: two plants received the chewing playback, while two received a sham control (silent actuator attached to the leaf). Caterpillar feeding vibrations were played back to plants for 2 h, as described above. Immediately after the playback, we allowed fourth instar *P. rapae* caterpillars to feed in individual clip cages (Electronic Supplementary Material Fig. 1B) on the vibrated leaf and an age-matched non-vibrated leaf (Fig. 1b) on all plants until approximately 30 % of the leaf area was consumed. At 24 and 48 h later, target and non-target leaves and the central group of unexpanded leaves were removed, flash frozen in liquid nitrogen and stored at −80 °C. Leaves were freeze-dried before analysis of GSs. We evaluated the influence of vibration treatment (chewing vibration vs. no-vibration control), tissue (playback leaf, same-age systemic leaf, unexpanded leaves in center of rosette) and sampling interval (24 vs 48 h) on the concentration of aliphatic and indolyl glucosinolates using a general linear mixed model in SAS v. 9.3 (using PROC GLIMMIX; SAS code provided in Electronic Supplementary Material, Table 1) with a gamma distribution (Bolker et al. 2009). Note that though we sampled the central rosette leaves on fed-upon plants to assess any changes in GS levels, these leaves were not themselves fed upon (it is not feasible to attach clip cages to these unexpanded leaves), and any changes seen in the rosette center would be a response to herbivory on other leaves. The four plants given the vibration treatment at the same time were treated as a block, and the block was included in the model as a random effect. Because we tested two response variables (total aliphatic glucosinolates and total indolyl glucosinolates), resulting *p* values were adjusted for multiple comparisons using the False Discovery Rate procedure (Benjamini and Hochberg 1995; Garcia 2004).

### Experiment #2

The design of this experiment was similar to the previous experiment, but with three differences that allowed us to answer additional questions. First, we asked whether vibrations elicited the production of phenolics, the second major class of defenses in *A. thaliana*, rather than glucosinolates. Second, we assessed the roles of direct induction (increased defenses in the absence of herbivory) vs. priming (increased defenses in response to herbivory), by adding a ‘no herbivory’ treatment consisting of plants that received the vibration playbacks and clip cages but no caterpillar feeding. Third, we asked whether the response to vibration was specific to chewing vibrations, as opposed to being induced by simply any vibration, a possibility not excluded by the first experiment. To address this question, we included two additional vibration controls: wind-induced vibrations, a common source of vibrational noise in the field; and the vibrational mating song of a leafhopper, chosen because it has a similar frequency spectrum to that of chewing, but a contrasting temporal pattern. Wind-induced vibrations were obtained by directing a small fan at *A. thaliana* plants of the same size as the experimental plants, and recording the leaf motion using laser vibrometry. Leafhopper recordings were drawn from a library of signals previously recorded by Cocroft.

As above, the amplitude of each chewing playback was matched to that of the original recording. The displacement amplitudes of the wind and leafhopper exemplars in each replicate were matched to that of the corresponding chewing exemplar. Chewing and leafhopper vibrations were played back using piezoelectric actuators, but the wind recordings contained mostly very low frequencies that were better replicated with a magnet and coil extracted from audio speakers (see Cocroft 2010 for a discussion of playback methods). To achieve uniform contact between all actuators and the playback leaf, short lengths of balsa dowel were attached to each transducer (Electronic Supplementary Material Fig. 1A), with the contact between dowel and leaf secured using accelerometer mounting wax. We conducted 18 replicates of the playbacks, each containing eight plants, with two plants per vibration treatment. The vibration treatments included caterpillar chewing, wind-induced vibrations, leafhopper mating song, and a sham with no vibration, as described above. Each replicate used different exemplar recordings (i.e., no recording was used in more than one replicate), and replicate was treated as a block for statistical analysis. The use of two plants for each treatment within a block allowed us to test for both direct induction and priming, by exposing half of the plants in each vibration treatment to caterpillar feeding and half to empty cages. To test for a direct effect, we measured defense chemistry 48 h after herbivory in playback and same-age systemic leaves of plants that experienced the vibration treatments but no herbivory. To isolate priming effects from direct effects, we took the ratio of the responses of the plants in the same replicate that did and did not experience herbivory. Because the matched plants received the same vibration exemplars, any direct effects will appear in both the numerator and denominator of the ratio, canceling out and leaving only the priming effect. Note that, as in Experiment #1, the unexpanded central rosette leaves did not experience herbivory.

We analyzed the data using a general linear mixed model with a gamma distribution, as above, to examine the influence of vibration treatment (chewing, wind, insect song, no vibration control) and tissue (vibrated leaf, non-vibrated same-age leaf, unexpanded leaves in the center of the rosette) on phenolic responses. Replicate (the set of eight plants tested at the same time) was included as a random block effect. Because we measured three response variables (anthocyanins, flavonoids, and phenolic redox activity as measured by the Folin-Denis assay), we used the false discovery rate procedure as described above to adjust the experiment-wide Type I error rate.

Note: the data from this study are provided in the Electronic Supplementary Material 2.

## Results

**Fig. 2:**
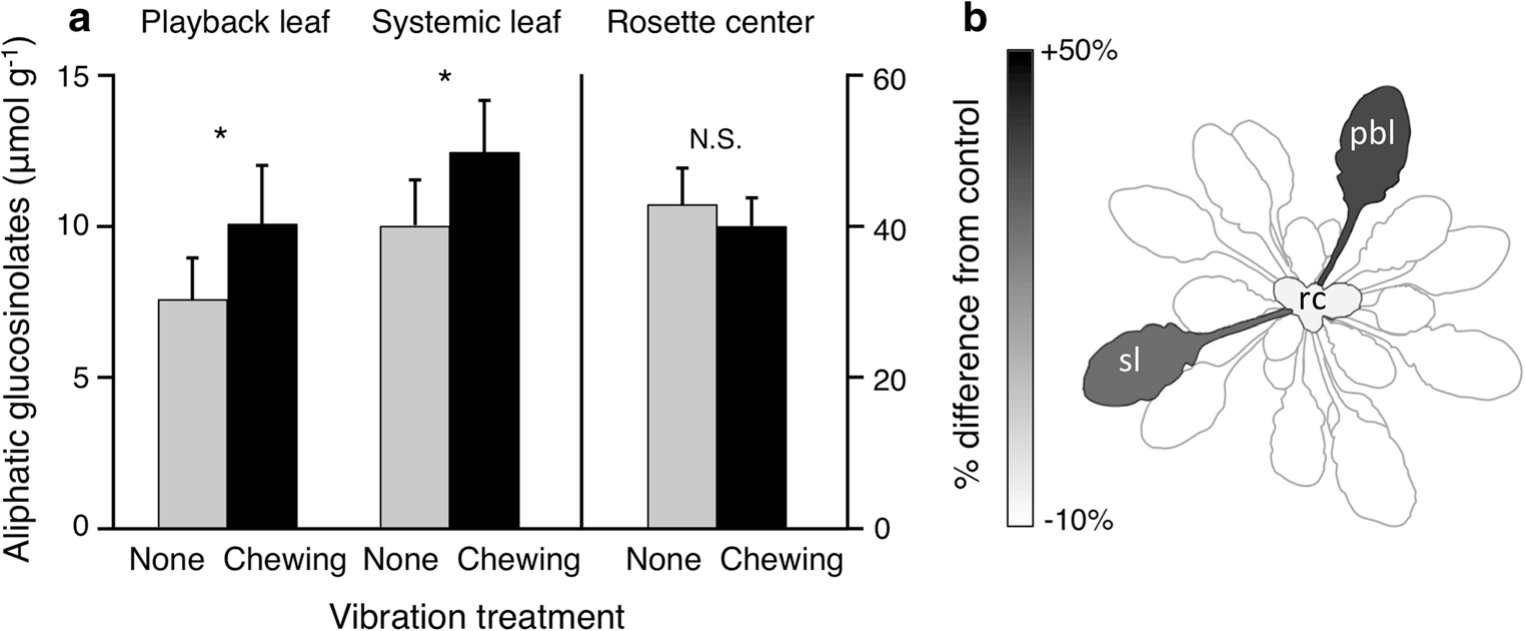
**a** Playback of caterpillar feeding vibrations increased the induced response of *A. thaliana* to herbivore damage, compared to no-vibration controls (*p < 0.05, *error bars* 95 % confidence intervals; there was no difference between the 24 and 48 h samples, so they were pooled here). *N* = 44 per bar (43 for rosette center). **b** Grayscale map showing the increase in aliphatic glucosinolates in the playback and same-age systemic leaves, expressed as the percent change from the levels in controls

Aliphatic GSs were higher in plants that had previously experienced chewing vibrations, than in plants that had experienced no vibrations (*p* < 0.05; Fig. 2a; Electronic Supplementary Material Table 1; note that all *p* values reported in the Results section have been adjusted for multiple comparisons using the False Discovery Rate procedure) and leaf type (*p* < 0.001). The levels of aliphatic GSs varied with leaf type, with glucosinolate levels higher in the unexpanded central rosette leaves than in the more mature leaves (*p* < 0.001; Fig. 2). Because there was a trend for the interaction of vibration treatment and leaf type (*p* < 0.07), we examined the response of the three leaf types from the same plant (the leaf receiving the playback; another leaf of the same age; and the unexpanded leaves in the center of the rosette). The response was both local and systemic, with similar changes in the vibrated leaf and a second leaf of the same developmental stage (Fig. 2a, both *p* < 0.05 based on post hoc comparisons). Aliphatic glucosinolates increased by 32 % in the playback leaf and 24 % in the same-age systemic leaf (Fig. 2b). There was no response in the unexpanded central rosette leaves (*p* = 0.59), which were not fed upon by the herbivore. There was no change in response to vibration treatment in indolyl glucosinolates (vibration treatment *p* = 0.71), although there was a nonsignificant trend for an interaction of leaf type and vibration treatment (Electronic Supplementary Material Table 2), and the changes in some individual indolyl compounds mirrored those for aliphatic compounds (Electronic Supplementary Material Fig. 3).

**Fig. 3:**
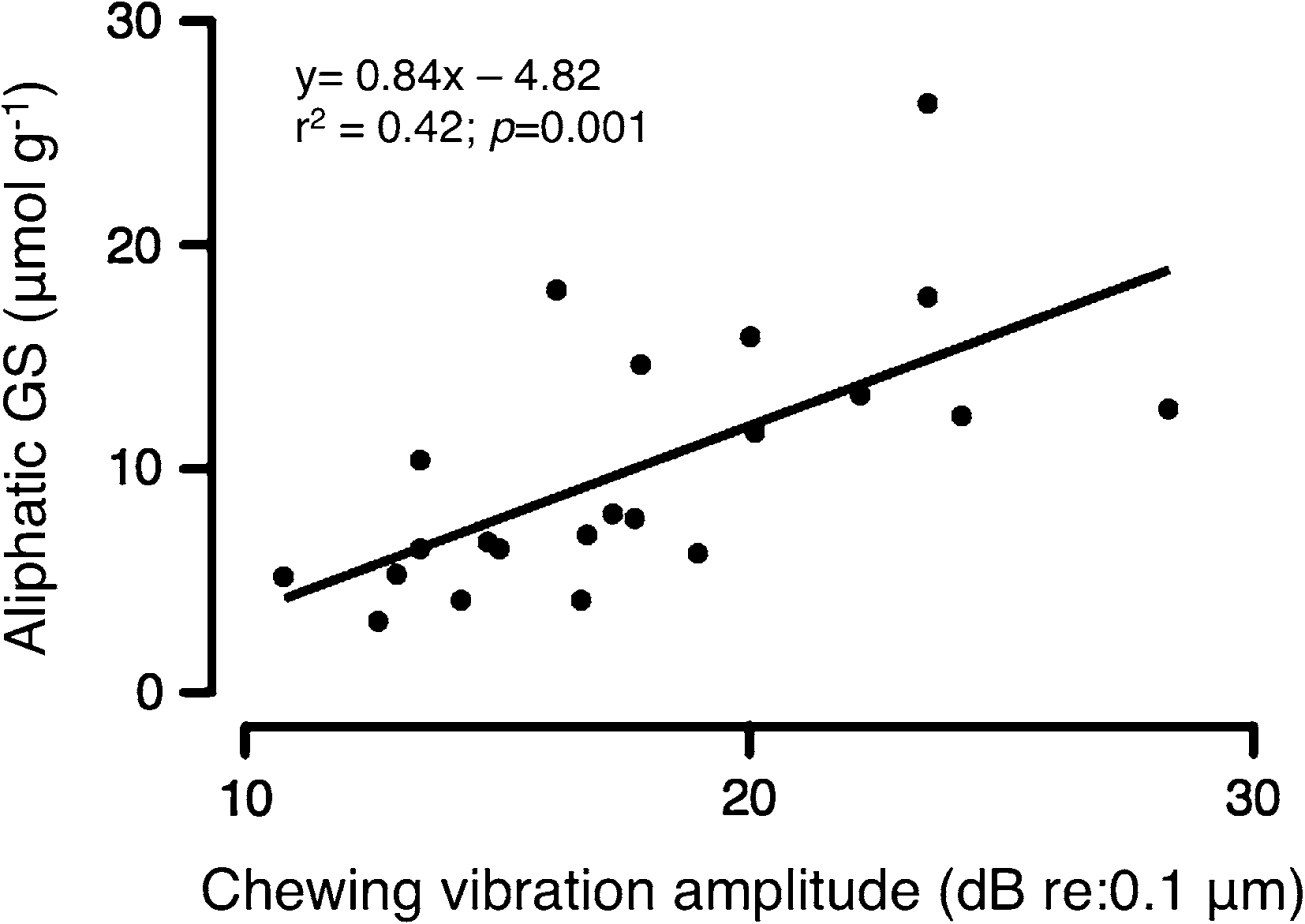
Relationship between the amplitude of the chewing vibration exemplars used in playbacks and the level of induced aliphatic GS. Linear regression, *N* = 22

Variation in amplitude among the 22 chewing exemplars influenced the glucosinolate response. In the recordings used for playback, the maximum displacement of the leaf surface caused by caterpillar chewing varied over nearly an order of magnitude (0.35–3.1 μm). Larger displacements caused the induction of more aliphatic GSs (averaging the 24 and 48 h samples for each exemplar) in the leaf receiving the playback (Fig. 3). The relationship was stronger when the displacement was expressed on a decibel scale than when expressed on a linear scale (*r*2 = 0.42 vs. 0.23).

**Fig. 4:**
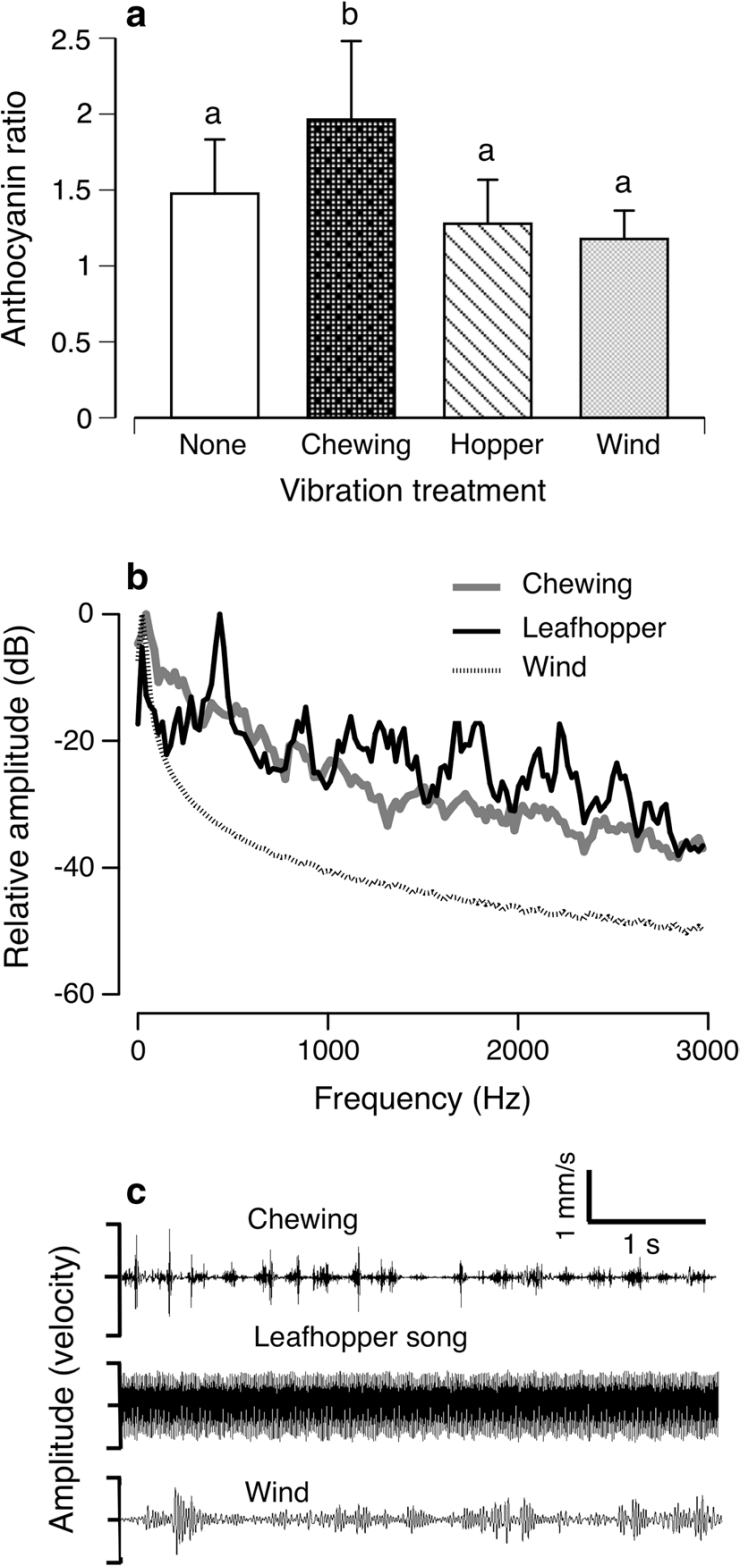
**a** Chewing vibrations increased the anthocyanin response to herbivory by *A. thaliana* ( the ratio of response in fed-upon plant vs. non-fed-upon plant, when both had same treatment exemplar). *Error bars* 95 % CI. *Letters above bars* indicate that the response to the chewing treatment was significantly different (*p* < 0.001) from responses to the three controls. **b** Averaged amplitude spectra of the stimuli used in the experiment (*N* = 18 for each stimulus type) suggest that chewing can be distinguished from wind, but not from leafhopper song, based on the frequency content. **c** Vibration waveforms of a chewing *P. rapae* caterpillar on *A. thaliana*; wind on *A. thaliana*; and a leafhopper, recorded on another host plant. Chewing and leafhopper song have similar amplitude spectra but different temporal features

In the second experiment, there was no direct induction caused by the vibration treatments—i.e., no increase in defenses in the absence of herbivory—for any of the polyphenol chemical defenses measured (anthocyanins, flavonoids or phenolic redox activity as measured by the Folin-Denis assay; all *p* > 0.37, Electronic Supplementary Material Fig. 4, Tables 3–5). However, there was a significant priming effect of the vibration treatment on the levels of anthocyanins after herbivory (vibration treatment, *p* < 0.05, Fig. 4; Electronic Supplementary Material Table 6). A planned contrast revealed that anthocyanin levels were significantly higher in plants that were pre-treated with chewing vibrations, than in the control treatments (vibrations from wind or leafhopper, no vibrations; Fig. 4a). There was an effect of leaf type (*p* < 0.05), with baseline levels of anthocyanins higher in the mature leaves than in the unexpanded central rosette leaves, but there was no interaction between leaf type and vibration treatment (*p* > 0.25). Flavonoids and phenolic redox activity were not primed by the vibration treatment (*p* > 0.7; Electronic Supplementary Material Fig. 5, Tables 7, 8).

Vibrations caused by caterpillar chewing were distinct from those caused by wind and leafhopper song (Fig. 4b, c). *P. rapae* caterpillars typically begin feeding on the leaf edge, producing a semicircular hole that enlarges as the caterpillar removes thin strips along the edge of the cut (to watch this behavior and listen to the vibrations produced, see Electronic Supplementary Material Fig. 6 for QR code and link to video). A strip of leaf tissue is removed as the caterpillar extends its head and gradually draws it closer to the body, closing its mandibles 3–5 times per second (*x* = 4.1 ± 0.56/s, *N* = 22 caterpillars). Each mandible closure produces a short pulse of vibrations (Figs. 1, 4c, Electronic Supplementary Material Fig. 2A) with a broad frequency range (Fig. 4b, Electronic Supplementary Material Fig. 2B). The vibration waveform thus consists of series of broadband pulses, with short pauses while the caterpillar extends its head to remove the next strip of tissue. The vibrations generated by low-velocity wind lack the high frequencies produced by chewing (Fig. 4b, c). Leafhoppers produced vibrational signals with a frequency spectrum similar to that produced by caterpillar chewing, but with a different temporal pattern (Fig. 4b, c).

## Discussion

Insects are among the most important consumers of plants in terrestrial ecosystems, and plants have evolved an array of traits that allow them to detect and respond to damage from feeding insects (Karban and Baldwin 1997). Changes in plants that increase resistance to subsequent herbivory can occur locally at the site of damage, or systemically at sites distant from the attack (Karban and Myers 1989). Localized feeding causes induction of chemical defenses in undamaged tissues by several proposed mechanisms, including signaling molecules that move within the plant (Pearce et al. 2008), airborne transport of leaf volatiles (Frost et al. 2007, 2008; Heil and Silva Bueno 2007a), and electrical signals (Mousavi et al. 2013; Fromm and Lautner 2007; Van Bel et al. 2011). Vibrational signals are likely to complement other signals that plants receive from herbivory; however, none of these mechanisms has been shown to transmit signals to all plant parts as rapidly as mechanical vibrations (10–100 m/s; Cocroft and Rodriguez 2005). We have found that plants can take advantage of this rapid and overlooked source of information about herbivory to produce a systemic response, via perception of the mechanical vibrations produced by feeding.

Glucosinolate and anthocyanin responses were both elicited by exposure to chewing vibrations, but at least in anthocyanins there was no direct effect; instead, anthocyanins were primed, with the increase in defenses revealed only as a response to herbivory. Was the induction of defenses large enough to have an ecological effect? For the noctuid caterpillar *Spodoptera exigua* feeding on *A. thaliana*, there is a strong negative correlation (*r* = −0.719) between induced glucosinolates and caterpillar growth rate (Mewis et al. 2005, Fig. 8F). We estimate from that relationship that the increase in total GS induction caused by exposure to chewing vibrations would decrease *S. exigua* growth rate by approximately 15–20 % (estimated from the levels of aliphatic + indolyl GS in the playback and same-age leaves, compared between control and chewing treatments). We lack similar information for the relationship between anthocyanins and *S. exigua* growth, so we cannot estimate the impact of increased anthocyanins on herbivores. Anthocyanins and other polyphenols have biological activity in many insects, but the effect varies, as with all putative chemical defenses, with the specific plant and herbivore combination (see Appel 1993 and Lattanzio et al. 2006 for reviews). For both kinds of chemistry, more precise estimates of their impact on fitness will require measuring vibration effects on induced defenses and insect growth, and examining other traits influencing plant fitness, such as oviposition choice.

Priming is “preparing for another battle” (Frost et al. 2008), a form of defense that prepares a plant to respond more quickly or more strongly to future herbivory. Under what circumstances might chewing vibrations predict a future herbivore attack? The most important role of herbivore vibrations is likely to be within individual plants, with vibrations propagating out from the fed-upon leaf, complementing other signal pathways to cause a systemic response. However, ‘eavesdropping’ between plants may be possible, as with the green leaf volatiles that function in within-plant signaling but can be perceived by neighboring plants (Karban et al. 2006; Heil and Silva Bueno 2007a, b). Vibrations can travel from plant to plant through connecting roots or stems (Cokl and Virant-Doberlet 2003), and even through the air between leaves that are within a few centimeters (Eriksson et al. 2011). Under those conditions, the vibrations generated by a chewing caterpillar could alert nearby plants to the presence of an herbivore. The observed relationship between vibration amplitude and induced glucosinolates suggests that the effect of herbivore vibrations will be largest near the source. However, although the amplitude of plant-borne vibrations decreases with distance, the decrease is not monotonic (Cokl and Virant-Doberlet 2003), and the same level of vibrational energy will cause more motion in smaller-diameter structures. Accordingly, understanding the within-plant and between-plant spread of vibration-induced defenses will require more precise mapping of the transmission of vibrational energy, and assessing the effectiveness of chewing vibrations in eliciting defense when their amplitude and other characteristics have been altered with distance. Furthermore, although *A. thaliana* responded differently to chewing vibrations, leafhopper song, and wind when each was presented alone, we do not yet know how the presence of multiple vibration sources influences signal detection. For example, does wind interfere with plant perception of herbivore vibrations, such that vibration-based herbivore detection functions best when wind speeds are low? Clearly, research on the sensory ecology of plants in a natural vibrational environment is needed to reveal the role of vibrations in plant defense in the field.

For a vibration-based herbivore detection system to function in nature, plants must distinguish the vibrations that signal herbivore feeding from the many environmental vibrations that do not. The priming of defenses in *A. thaliana* plants is indeed selective: anthocyanins were primed by caterpillar chewing vibrations, but not by wind vibrations or leafhopper song. How such selectivity is achieved is an open question in plant sensing. Acoustically signaling animals, such as frogs and insects, distinguish among signals on a multivariate basis, rather than a univariate one (Gerhardt and Huber 2002). The selective responses of the *A. thaliana* plants in this study may also have depended on a combination of signal features. For example, chewing and wind-induced vibrations differ greatly in their frequency content: chewing vibrations contain both low and high frequencies, whereas wind vibrations are dominated by low frequencies (Fig. 4b; Cocroft and Rodriguez 2005). Rootlets of *Z. mays* seedlings grow more strongly toward waterborne tones in a particular frequency range (Gagliano et al. 2012a). Likewise, it would be possible for *A. thaliana* leaves to selectively prime their defenses to chewing by responding only to vibrations containing higher frequencies. However, although frequency range may be sufficient for *A. thaliana* to distinguish chewing from wind-induced vibrations, a frequency-based mechanism is unlikely to account for the plants’ lack of response to leafhopper song, whose amplitude spectrum is broadly overlapping with that of chewing (Fig. 4b). Chewing vibrations and leafhopper song do differ strikingly, however, in their temporal pattern: chewing vibrations consist of repeated, short bursts of energy, while the leafhopper song was relatively constant in amplitude (Fig. 4a). The selectivity of *A. thaliana* may thus rely on both frequency content and gross-temporal features. Reliance on multiple signal components would likely increase the reliability of vibration-based herbivore detection, because vibrational signals are subject to frequency filtering and degradation of temporal features as they propagate along plant stems (Fig. 1; Virant-Doberlet and Cokl 2004).

The mechanisms used by plants to detect and respond to mechanical vibration have received a burst of recent experimental attention (Chehab et al. 2009; Monshausen and Gilroy 2009; Niklas 2009; Coutand 2010; Li and Gong 2011; Gagliano et al. 2012a, b; Veley and Haswell 2012; Haswell and Monshausen 2013; Gagliano and Renton 2013). Mechanoreception is thought to start by triggering of mechanosensors in the cell wall and/or plasma membrane, similar to those known from bacteria but unconfirmed in plants (Haswell and Monshausen 2013). The mechanosensors cause fluxes of Ca^2+^, ROS, and H^−^, which trigger downstream responses that involve many plant hormones, and rapid expression of genes that respond early to many plant stresses (Lee et al. 2005; Walley et al. 2007; Kagaya and Hattori 2009). Several of these hormones, especially jasmonates and ethylene and their respective biosynthetic pathways, have important roles in plant responses to herbivory (Moreno et al. 2009; Leon-Reyes et al. 2010). As a result, jasmonate and ethylene signaling pathways are a likely proximate mechanism by which plant-borne mechanical vibrations that mimic insect feeding influence early plant defense responses. Additional information about the specific vibrational cues used by *A. thaliana* may also inform the search for mechanisms; for example, the relationship between vibration amplitude and the glucosinolate response was stronger when amplitude was expressed on a decibel scale, suggesting a parallel between plant perception and animal perception (Varshney and Sun 2013) of mechanical stimuli.

The ability of plants to increase their defenses in response to micrometer-scale vibrations lends support to recent hypotheses that plants can detect and respond to low-amplitude vibrations produced by neighboring plants (Gagliano et al. 2012a, b, Gagliano and Renton 2013). Plant responses to acoustic cues were suggested by studies showing that seedling germination (Gagliano et al. 2012a) and growth (Gagliano et al. 2012b) are influenced by the presence of nearby plants, even when visual and chemical cues were excluded (Gagliano and Renton 2013). The above-ground and below-ground portions of plants will experience contrasting vibrational environments, with soil damping much of the vibrational energy originating in leaves and stems (Hill 2008). However, below-ground herbivory can be extensive (van Dam 2009), and the vibrations generated by root herbivores are also a potential set of cues for inducible defenses in roots.

The vibrations produced by chewing herbivores likely interact with other cues to elicit systemic defenses. The current study suggests two hypotheses, not mutually exclusive, for why playback of chewing vibrations caused systemic priming of glucosinolates and anthycyanins. First, because vibrations propagate throughout the plant, the transmitted vibrations could have caused priming in the systemic leaves, as they did in the playback leaf. Alternatively, the vibrations in the playback leaf could have triggered systemic signaling from that leaf in the form of airborne volatiles, phloem-borne signals, or electrical signals. If chewing vibrations do cause the release of airborne volatiles, it is possible that vibrational and volatile signaling could have a synergistic effect as they do in social insects (Hölldobler 1999), where both function to communicate alarm within a colony and each can modulate the threshold of response to the other. Future research will be designed to understand how mechanical vibrations interact with other forms of within-plant information transfer to generate systemic responses to herbivory.

## Electronic supplementary material

Below is the link to the electronic supplementary material.

Supplementary material 1 (DOCX 2068 kb)

Supplementary material 2 (XLS 163 kb)
