# Supplementary material for: Plants respond to leaf vibrations caused by insect herbivore chewing
Source: Oecologia. 2014 Jul 2;175(4):1257–66. doi: 10.1007/s00442-014-2995-6 (PMC4102826; doi:10.1007/s00442-014-2995-6)
Supplement: Supplementary file 1 — Supplementary material 1 (DOCX 2068 kb) [file 442_2014_2995_MOESM1_ESM.docx]

**Article title:** **Plants respond to leaf vibrations caused by insect herbivore chewing**

**Authors:**

**H. Appel**

Division of Plant Sciences and Bond Life Sciences Center, University of Missouri, Columbia MO, 65211

Email: appelh@missouri.edu.

**R. B. Cocroft**

Division of Biological Sciences, University of Missouri, Columbia MO, 65211

Email: [cocroftr@missouri.edu](mailto:cocroftr@missouri.edu)

Note: The data sets for this study are included in a separate document (ESM_2.xls)

**Figure 1**. Equipment used for vibrational playbacks and subsequent herbivore feeding on experimental plants. **A**. Vibrational playback setup, showing a piezoelectric actuator (green, upper left) and a modified speaker (lower right). Both were connected to the playback leaf using a balsa-wood dowel with accelerometer mounting wax at the tip. **B**. Clip cages used to confine feeding caterpillars to individual leaves; two leaves were sampled for chemistry and one leaf was sampled for future gene expression studies. Cages contain a mesh window for observing caterpillar feeding.

**Figure 2**. Recordings of original and played-back chewing vibrations of a *P. rapae* caterpillar on *A. thaliana*. **A**: Waveform of the vibrations produced by the feeding caterpillar (above), and of the same vibrations played back with a piezoelectric actuator, after filtering that compensates for the frequency response of the playback system. **B**: Amplitude spectra of the original and played-back vibrations shown in **A**.

**Figure 3**. Response of glucosinolates to playback of chewing vibrations, for the playback leaf (pbl), same-age systemic leaf (sl), and the rosette center of young unexpanded leaves (rc). Aliphatic glucosinolates are shown in A-E, and indolyl glucosinolates in F-H. Note that the indolyl compounds I3M (F) and 1MOI3M (H) show the same pattern as the aliphatic compounds in A, B, and E, with increases in the chewing treatment in the playback and same-age systemic leaves and a slight decrease in the rosette center. This pattern is reflected in a non-significant trend (.05<p<0.1) for an interaction of leaf type and treatment for the total indolyl glucosinolates (see main article). Error bars = 95% CI. Abbreviations: 3MSOP, 3-methylsulfinylpropyl-glucosinolate; 4MSOB, 4-methylsulfinylbutyl glucosinolate; 6MSOH, 6-methylsulfinylhexyl glucosinolate; 4MTB, 4-methylthiobutyl glucosinolate; 8MSOO, 8-methylsulfinyloctyl glucosinolate; I3M, indol-3-yl-methyl glucosinolate; 4MOI3M, 4-methoxy-indolyl-3-methyl glucosinolate; 1MOI3M, 1-methoxy-indolyl-3-methyl glucosinolate. Two additional glucosinolates were tested for but not detected: 5MSOP, 5- methylsulfinylpentyl glucosinolate; and 7MSOH, 7-methylsulfinylheptyl glucosinolate.

**Figure 4**. Measurement of defense levels in plants treated with vibrational playback but not subjected to herbivory. There was no direct effect of vibration treatments on anthocyanins (A), flavonoids (B), or total redox-reactive phenolics (C). Error bars = 95% CI. See Tables 3-5 below for statistical tests.

**Figure 5**. There was no priming effect of vibration treatment on flavonoids (A) or total redox-reactive phenolics (B); Y-axis shows the ratio of response in attacked plant vs. non-attacked plant, when both had same treatment exemplar. Error bars = 95% CI. See Tables 6-8 below for statistical tests, and main article for the figure showing the priming of anthocyanins by the chewing vibration treatment.

**Figure 6**. QR code for online video of a *P. rapae* caterpillar feeding on a leaf of *A. thaliana*. The audio track for this video is a laser vibrometry recording of the vibrations produced by the feeding caterpillar. The video can also be viewed via this link: <https://www.youtube.com/watch?v=ndfo2NiaEIE&feature=youtu.be>

**Table 1.** General linear mixed model testing for the priming effect on **aliphatic glucosinolates** of vibration treatment (chewing vs silent control), leaf type (playback leaf, same-age systemic leaf, rosette center), time interval between vibration treatment and herbivory (24h vs 48 h), and their interactions*. Replicate was included as a random block effect. The raw p-values are presented here, and those in bold remain significant after correcting for the experiment-wide False Discovery Rate (the FDR correction was used because two dependent variables were tested -- aliphatic and indolyl glucosinolates -- and thus there were two ‘treatment’ effects requiring adjusted p-values, two ‘leaf type’ effects, etc.).

effect df F p

treatment 1,229 6.06 **0.0146**

leaf type 2,229 267.65 **<0.0001**

treatment x leaf type 2,229 2.9 0.0573

interval 1,229 0.10 0.7488

treatment x interval 1,229 0.00 0.9724

interval x leaf type 2,229 2.28 0.1050

treatment x interval 2,229 0.51 0.5994

x leaf type

*SAS code:

PROC GLIMMIX;

CLASS REPLICATE TREATMENT INTERVAL TISSUE;

MODEL {DEPENDENT VARIABLE}=TREATMENT|TISSUE|INTERVAL /DIST=GAMMA LINK=LOG SOLUTION;

RANDOM INT / SUBJECT=REPLICATE;

RUN;

Note: this model uses the conservative ‘containment’ option for the denominator degrees of freedom (DDFM), and is robust to other methods of estimating the DDFM.

**Table 2.** General linear mixed model testing for the effect on **indolyl glucosinolates** of vibration treatment (chewing vs silent control), leaf type (playback leaf, same-age systemic leaf, rosette center), interval between vibration treatment and herbivory (24 vs 48 h), and their interactions. Replicate was included as a random block effect. p-values reported as in Table 1.

effect df F p

treatment 1,229 6.06 0.7088

leaf type 2,229 267.65 **<0.0001**

treatment x leaf type 2,229 2.9 0.0651

interval 1,229 0.10 0.5307

treatment x interval 1,229 0.00 0.5163

interval x leaf type 2,229 2.28 0.6526

treatment x interval 2,229 0.51 0.6132

x leaf type

**Table 3**. General linear mixed model testing for direct induction of **anthocyanins** by vibration (measured in plants that experienced the vibration treatments but no herbivory). Effects include vibration treatment (silent control, chewing, leafhopper song, wind), leaf type (playback leaf, same-age systemic leaf, rosette center), and their interaction. Replicate was included as a random block effect. SAS model as in footnote to Table 1. p-values reported as in Table 1.

effect df F p

treatment 3,140 1.37 0.2557

leaf type 2,140 43.68 **<0.0001**

treatment x leaf type 6,140 0.79 0.6513

**Table 4**. General linear mixed model testing for direct induction of **flavonoids** by vibration (measured in plants that experienced the vibration treatments but no herbivory). Effects include vibration treatment (silent control, chewing, leafhopper song, wind), leaf type (playback leaf, same-age systemic leaf, rosette center), their interaction. Replicate was included as a random block effect. SAS model as in footnote to Table 1. p-values reported as in Table 1.

.

effect df F p

treatment 3,139 1.44 0.2344

leaf type 2,139 69.61 **<0.0001**

treatment x leaf type 6,139 0.61 0.7195

**Table 5**. General linear mixed model testing for direct induction of **total redox-reactive phenolics, as measured using the Folin-Denis assay,** by vibration (measured in plants that experienced the vibration treatments but no herbivory). Effects include vibration treatment (silent control, chewing, leafhopper song, wind), leaf type (playback leaf, same-age systemic leaf, rosette center), their interaction. Replicate was included as a random block effect. SAS model as in footnote to Table 1. p-values reported as in Table 1.

effect df F p

treatment 3,140 0.91 0.4356

leaf type 2,140 27.18 **<0.0001**

treatment x leaf type 6,140 0.81 0.5653

**Table 6**. General linear mixed model testing for the priming effect on **anthocyanins** (expressed as the ratio of anthocyanin level in the fed-upon plant to that in the non-fed-upon plant, when both were in the same replicate and received the same vibration exemplars). Effects include vibration treatment (silent control, chewing, leafhopper song, wind), leaf type (playback leaf, same-age systemic leaf, rosette center), their interaction. Replicate was included as a random block effect. p-values in bold are those that remain significant after correcting for the experiment-wide False Discovery Rate (the FDR correction was used because three dependent variables were tested: anthocyanins, flavonoids, and total redox-reactive phenolics). SAS model as in footnote to Table 1.

effect df F p

treatment 3,129 4.24 **0.0068**

leaf type 2,129 5.33 **<0.006**

treatment x leaf type 6,129 1.33 0.2489

**Table 7**. General linear mixed model testing for the priming effect on **flavonoids** (variable is a ratio as described in Table X). Effects include vibration treatment (silent control, chewing, leafhopper song, wind), leaf type (playback leaf, same-age systemic leaf, rosette center), their interaction. Replicate was included as a random block effect. p-values reported as in Table 6.

effect df F p

treatment 3,126 0.37 0.7722

leaf type 2,229 267.65 <0.1684

treatment x leaf type 2,229 2.9 0.2758

**Table 8**. General linear mixed model testing for the priming effect on **total redox-reactive phenolics, as measured using the Folin-Denis assay** (variable is a ratio as described in Table X). Effects include vibration treatment (silent control, chewing, leafhopper song, wind), leaf type (playback leaf, same-age systemic leaf, rosette center), their interaction. Replicate was included as a random block effect. p-values reported as in Table 6.

effect df F p

treatment 3,127 0.51 0.673

leaf type 2,127 0.30 <0.7382

treatment x leaf type 6,127 1.14 0.3419
